# Shape of You: Eye-Tracking and Social Perceptions of the Human Body

**DOI:** 10.3390/bs15060817

**Published:** 2025-06-14

**Authors:** Edward Morrison, Marianne Lanigan

**Affiliations:** Centre for Comparative and Evolutionary Psychology, University of Portsmouth, Portsmouth PO1 2UP, UK

**Keywords:** eye-tracking, BMI, attractiveness, youthfulness, healthiness, bodies, social perception

## Abstract

Much research has considered how physical appearance affects the way people are judged, such as how body size affects judgements of attractiveness and health. Less research, however, has looked at visual attention during such judgements. We used eye-tracking to measure the gaze behaviour of 32 participants (29 female) on male and female computer-generated bodies of different body mass index (BMI). Independent variables were sex and BMI of the model, area of interest of the body, and the judgement made (attractiveness, healthiness, and youthfulness). Dependent variables were the number and duration of fixations, and Likert ratings. Most visual attention was paid to the chest and midriff, but this pattern differed slightly depending on the judgement being made, and on the BMI of the body. The sex of the body also affected eye-gaze behaviour, possibly because most participants were female. The bodies at the lower end of healthy weight were judged most attractive and healthy, in line with previous research, but the lightest bodies were judged as most youthful. These results suggest that these social judgements cue similar but subtly different gaze behaviour, and broadly support the “health-and-fertility” hypothesis, that the most attractive bodies are those that indicate evolutionary fitness.

## 1. Introduction

Humans tend to make rapid, automatic judgements based on physical appearance. For instance, facial impressions form within milliseconds and remain consistent even with extended viewing time (Willis & Todorov, 2006). Features such as facial attractiveness, expression, and race significantly influence these social perceptions (Rule & Ambady, 2010; Hack, 2014; Trent & Ferguson, 2021). Similarly, body shape and size, particularly body mass index (BMI), affect how individuals are perceived. According to the UK National Health Service (2023), BMI scores between 18.4 and 24.9 are healthy, while those lower are underweight, those between 25 and 29.9 are overweight, and scores between 30 and 39.9 are obese, with lower ranges for people of Asian or black ethnicity. In Western cultures, lower BMI is generally associated with attractiveness, success, and discipline, whereas higher BMI is often linked to negative stereotypes such as laziness and lack of intelligence (Puhl & Heuer, 2009; Van Amsterdam, 2013). Less research, however, has looked at visual attention during such judgements. This study addresses that gap by using eye-tracking to examine how people visually assess computer-generated bodies of varying BMIs while making judgements of attractiveness, healthiness, and youthfulness.

### 1.1. Body Size and Attractiveness

From an evolutionary standpoint, physical traits can serve as signals of biological quality. Judgements of attractiveness may reflect evolved preferences for traits indicating health and reproductive fitness. In women, there appears to be an inverted U-shaped relationship between BMI and perceived attractiveness, with peak attractiveness reflecting optimal fertility (Tovée et al., 1999a) and health (see Section 1.2).

By contrast, men’s attractiveness is less tied to BMI and more to muscularity and cues of social dominance (Kenrick et al., 1994; Hargreaves & Tiggemann, 2009; Brierley et al., 2016). While a lean figure is considered attractive in women, the ideal male body type is muscular, often translating into a higher BMI due to muscle mass rather than fat (Ahmadi et al., 2018). Cues such as wide shoulders (Sell et al., 2017; Tovée et al., 1999b) and general muscularity make male bodies more attractive (Frederick & Haselton, 2007). Men are often motivated to lose weight and gain muscle to match these body ideals (Drewnowski & Yee, 1987). Crossley et al. (2012) demonstrated this divergence using 3D body manipulation software (Daz Studio 3.1 from Daz3d.com): women preferred a female BMI around 18.9 and a male BMI around 24.5, while men preferred slightly leaner women and more muscular men, with male body preferences often exceeding the “healthy” BMI range due to muscle mass.

Cultural factors also play a significant role. Media exposure contributes to narrow ideals, particularly for women, where slimness is highly valued (Swami, 2015). For example, Boothroyd et al. (2020) found that higher television consumption was linked to preferences for slimmer female bodies, supporting the idea that media influences body ideals.

### 1.2. Body Size, Health, and Youth

Body size influences not only perceptions of attractiveness but also judgements of health and age. High BMI (overweight: 25–29.9, obese: 30–39.9) is associated with increased risk of diseases like coronary heart disease, type 2 diabetes, and non-alcoholic fatty liver disease (Katta et al., 2021; Wondmkun, 2020; Luo & Lin, 2021). Mental health and quality of life also decline with higher BMI, with associations found for anxiety, depression, and reduced physical functioning (Meixner et al., 2020; Yancy et al., 2002; Sharafi et al., 2020; Dowd & Zajacova, 2015). Indeed, high BMI is regarded as a major public health concern globally (Williams et al., 2015).

Conversely, extremely low body weight also has serious health implications, including anaemia, cardiovascular disease, infertility, and liver dysfunction due to malnutrition (Khan et al., 2018; Kwon et al., 2021; Boutari et al., 2020; Risi et al., 2021). In women, waist-to-hip ratio (WHR) and BMI have been linked to health and fertility (Lassek & Gaulin, 2016; Erel & Senturk, 2009; Singh, 2002), with very low BMIs (below 19) associated with reduced health and fertility (Rich-Edwards et al., 2002). The health-and-fertility hypothesis posits that physical attractiveness in women is partly a proxy for cues like hormonal status, health, and youth (Tovée et al., 1998; Tovée et al., 1999a). Media-driven thin ideals have contributed to a rise in underweight BMIs, especially among women (Bird et al., 2017; Mayer-Brown et al., 2016). Consequently, public perceptions of health are heavily influenced by body size, with moderate BMIs seen as healthier (Swami et al., 2007).

C. Han et al. (2023) tested the health-and-fertility hypothesis by examining if the BMI of the most attractive female body matched that of the most healthy-looking one. Interestingly, results showed that the most attractive models had significantly lower BMIs than the models rated as most healthy, challenging the hypothesis that attractiveness and health align perfectly.

Body size also affects age-related perceptions. Since BMI tends to rise with age, it may be used implicitly as a cue for age. Overweight and obesity peak between the ages of 50 and 74 (NatCen Social Research, University College London, Department of Epidemiology and Public Health, 2024), with similar trends observed using bioimpedance measurements (Meeuwsen et al., 2010). Supporting this, T. S. Han et al. (1999) found that silhouettes with increasing BMI were judged to be older. However, follow-up research in this area remains limited. From a Darwinian perspective, youthfulness is evolutionarily significant, especially in females. Reproductive value, the expected future reproductive output of an individual, declines with age, making youth an essential indicator of reproductive potential (Andrews et al., 2017). Because BMI typically increases with age, leaner bodies may be perceived not only as more attractive but also more youthful, thereby indirectly enhancing perceived reproductive value. This may help explain why individuals often prefer body types that are slightly slimmer than what is optimal for physical health. Evolutionary pressures may have shaped human preferences for physical cues of youthfulness to maximise reproductive success.

### 1.3. Eye-Tracking Attractiveness

Despite extensive research on how body size influences social judgements, relatively little is known about visual behaviour during these perceptions. Eye-tracking offers a valuable tool to explore this, as eye movement may be less susceptible to social desirability bias and may reflect automatic processes. Eye-tracking can reveal complementary evidence to explicit ratings. For instance, Dixson et al. (2011) examined men’s eye movements while viewing unclothed women of varying WHRs and breast sizes. Participants consistently fixated more on breasts than other body areas, particularly during initial fixations, suggesting these regions carry greater perceived value in attractiveness judgements.

Hewig et al. (2008) explored gender differences in gaze behaviour when viewing clothed male and female bodies. Both men and women looked primarily at faces, but men looked earlier and longer at women’s breasts, whereas women focused more on men’s legs. Cornelissen et al. (2009) investigated male and female observations of female attractiveness and how it was affected by body fat and WHR. Results showed that body fat fixations were similar to attractiveness fixations, suggesting that BMI strongly predicted attractiveness ratings.

### 1.4. The Present Study

Taken together, these findings suggest that body size, sex, and visual attention interact in complex ways when making social judgements. While the role of body size and shape on person perception has been well researched, relatively little research has used eye-tracking to measure perceptual behaviour and where on the body visual attention is paid. Those studies that have tend to use a single judgement type, often attractiveness, but not putatively related judgements such as youthfulness and healthiness. The aim of this study is similar to that of Dixson et al. (2011), in that we explore eye gaze behaviour on computer models of human bodies that vary in shape. The difference is that we include both male and female bodies that vary in BMI rather than WHR, and have participants make judgements not just of attractiveness but also of healthiness and youthfulness. Our primary hypothesis is that fixation duration and number of fixations will depend on model sex, BMI, and area of interest (AOI), and that these patterns will be different when assessing attractiveness, healthiness, and youthfulness. Our secondary hypothesis is that participants’ ratings of attractiveness, healthiness, or youthfulness will depend on model sex and model BMI.

## 2. Materials and Methods

A total of 32 participants (29 female, three male) were recruited opportunistically or by volunteering to participate through advertisement posters or an online participant pool in exchange for course credit. Most of the sample comprised university students studying psychology. The participants were between 18 and 42 years old (*M* = 19.83, *SD* = 4.61), showing that most of the sample were female students around 19. Furthermore, sexual orientation was also noted (if the participant chose to disclose this information), with 62% of female participants being heterosexual and 21% bisexual, and one male participant heterosexual and one bisexual (one did not disclose). Exclusion criteria were wearing natural false lashes or no false lashes at all, since this typically confuses the eye-tracking equipment, and age <18 years. Glasses were allowed, as the equipment had been tested with glasses and found fully operational, provided the lenses were kept clean.

### 2.1. Design

A 2 (model sex: female and male) by 5 (model BMI: underweight, lower-end healthy weight, higher-end healthy weight, overweight, and obese) by 5 (AOI: head, chest, midriff, thighs, and lower legs and feet) by 3 (rating type: attractiveness, healthiness, and youthfulness) within-subject repeated measures design was used for this study. For hypothesis 1, the dependent variables were fixation duration (how long the participant looked at an AOI) and number of fixations (how many times the participant focused on an AOI). For hypothesis 2, the dependent variables were the Likert rating scores, which were between 1 and 6 for three different rating types: (1) very unattractive/unhealthy/mature, (2) unattractive/unhealthy/mature, (3) somewhat unattractive/unhealthy/mature, (4) somewhat attractive/healthy/youthful, (5) attractive/healthy/youthful, and (6) very attractive/healthy/youthful.

### 2.2. Materials

Eye movements were recorded using the Tobii X2-30 Compact at a sampling rate of 30 Hz and a system latency of 50–70 ms. A laptop dedicated to this programme was set up alongside the primary Tobii eye-tracking camera, which sat below the screen towards the participant. We used a velocity threshold of 30 degrees/second and a minimum fixation duration of 60 msec. Adjacent fixations were merged if they were <75 msec and <0.5 degrees apart. No gap-fill interpolation was used (i.e., blink and saccades were not considered as fixations). The eye-tracker was calibrated with each participant before data collection using screen-based calibration. The participant looked at calibration targets at multiple locations on the screen to ensure that these data were automatically mapped to those locations using a standard configuration of the 3D eye model. Head position was fixed during the experiment to ensure eye-tracking data was robust.

**Figure 1:**
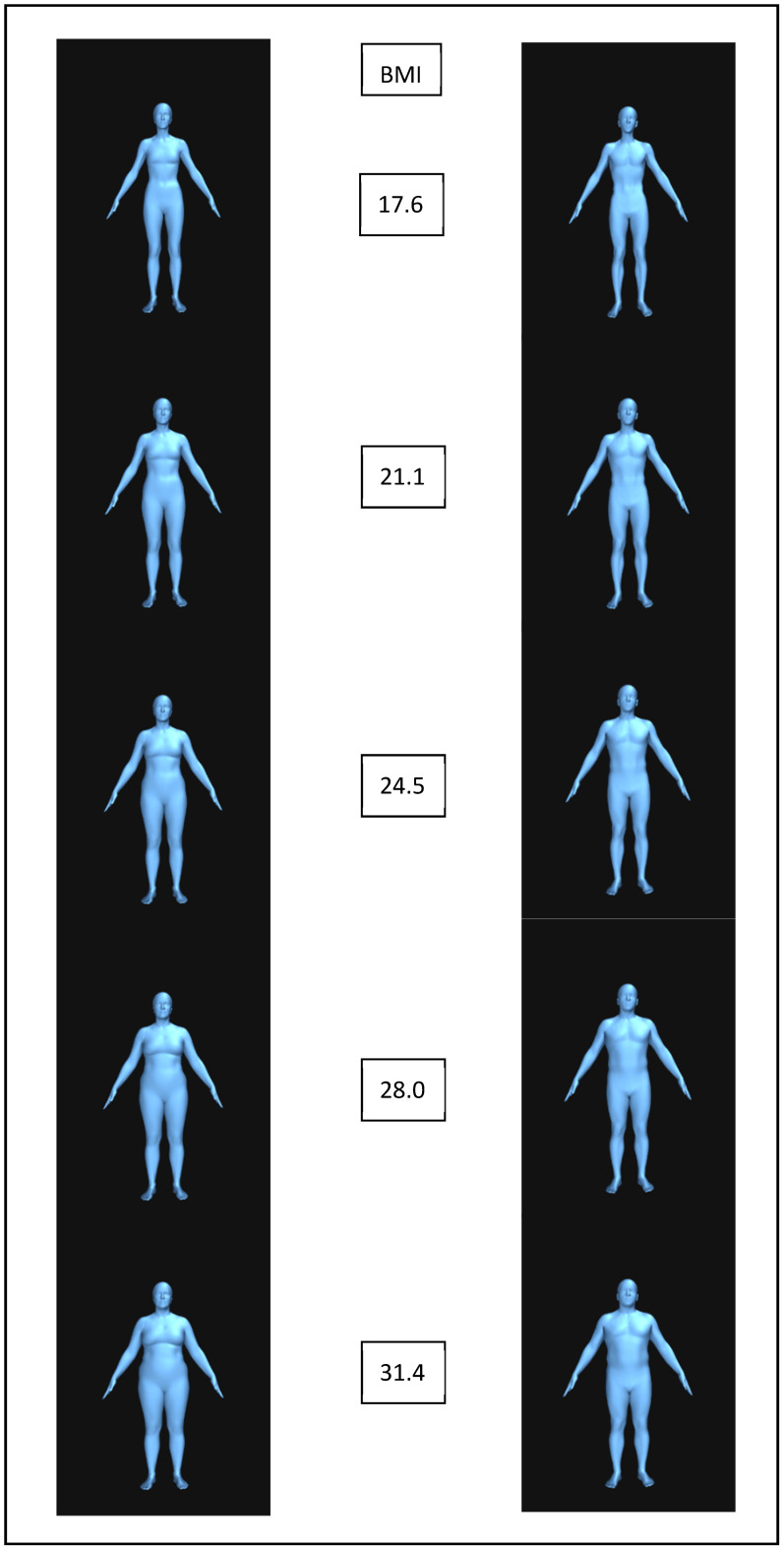
Female (**left**) and male (**right**) bodies with increasing BMI going downwards.

An online tool from the Max Planck Society (https://bodyvisualizer.is.tue.mpg.de/ accessed on 4 October 2023) was used to create male and female stimuli. For each sex, five stimuli were created across different BMI levels: underweight (51 kg, the lowest BMI allowed of 17.6); lower-end healthy weight (61 kg, BMI 21.1); higher-end healthy weight (71 kg, BMI 24.5); overweight (81 kg, BMI 28); and obese (91 kg, BMI 31.4). Figure 1 shows the female and male models. Models were kept at a height of 170 cm, with 7 h of exercise a week, to standardise them. However, all other measurements, such as the chest, waist, and hip measurements, were approximated by the tool to how they would realistically look with that weight.

**Figure 2:**
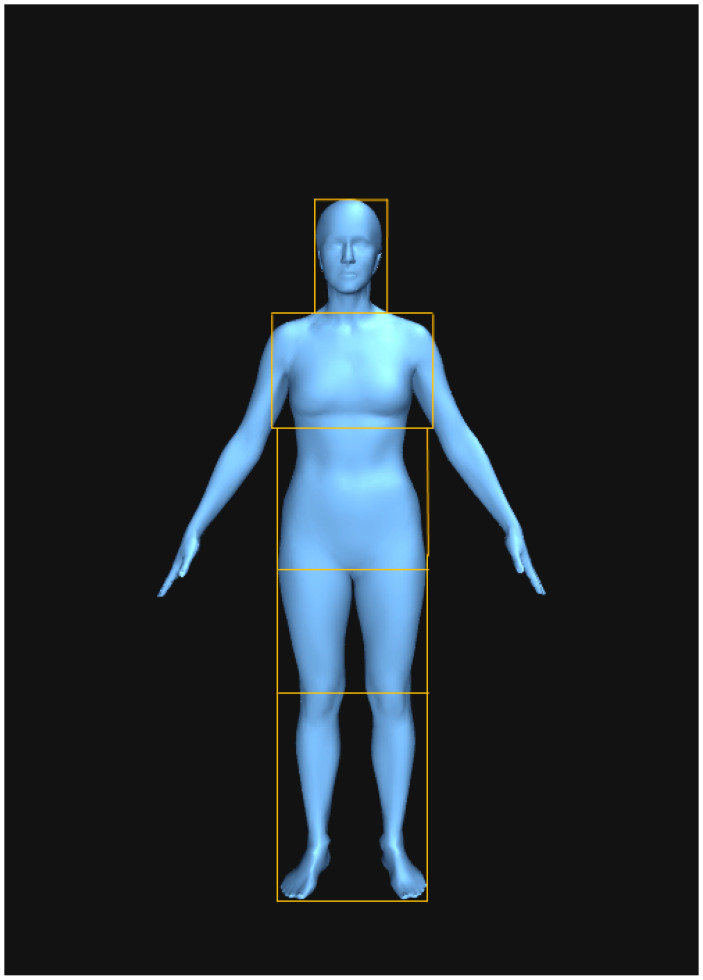
AOIs indicated on the higher-end healthy weight female model.

In Tobii Pro Lab (version 1.217), AOIs were defined from a similar study that investigated men’s preferences for WHR and breast sizes on women through eye-tracking (Dixson et al., 2011): head (the face and neck); chest (collarbone to directly under the chest); midriff (lower chest to the broadest part of the hips); thighs (widest part of the hips to slightly above the knee); lower legs and feet (above the knee to the feet). Dixson et al. (2011) also defined the pubic region as an AOI, but this was not necessary for this study since the models did not show genitalia (Figure 2).

Other materials were demographic questions, asking age, sexual orientation, gender/biological sex, and ethnicity; participants were not required to answer all demographic questions except for age if they did not wish to. Participants entered their ratings on a pencil-and-paper response sheet, which had six tables worth of 5 Likert scales, one for each of the images. Each table had cells to record ratings for attractiveness, youthfulness, and healthiness, with ratings ranging from 1 (very unattractive/unhealthy/mature) to 6 (very attractive/healthy/youthful).

### 2.3. Procedure

The study was ethically approved by the University of Portsmouth Ethics Committee and was deemed to have no substantial ethical issues. Before the study, participants were seated in a comfortable chair in a quiet laboratory room, facing the laptop at eye level at a distance of 60 cm. Following the completion of the participant information sheet and informed consent form, calibration was initiated by the researcher, who instructed the participant to follow a red dot across the screen with their gaze. This calibration was validated for gaze accuracy and precision, as per the guidance of the specification guide. Before testing began, participants were shown the response sheet and how to fill it in correctly. After that, testing began, and instructions were presented on the screen for the participant as follows:

*“Following these instructions, you will be presented with five female images. Please write down your attractiveness ratings of the following images on the response sheet. Please press Spacebar to continue.”*

Participants were then shown five images of the female model stimulus, ranging from underweight to obese BMI. The first image was presented for 5 s for the individual to examine, followed by a black screen showing the following instructions:

*“Record your answer. Please press Spacebar to continue.”*

After noting their perceived attractiveness rating of the model, they would examine the second image and repeat the same actions. This would continue until all five images were viewed, and then the participants would view the same images but rate for healthiness and again for youthfulness. Then, this would repeat for all of the male model stimuli. Following the study, participants would be debriefed verbally and thanked for participating. The presentation software did not have a function to randomise only specific images rather than all of them. Order of presentation of images was therefore counterbalanced, with participants assigned at random to either view the image from lightest to heaviest, or from heaviest to lightest. Participants rated attractiveness for each stimulus first, then repeated the viewings in the same order for rating of youthfulness, then healthiness.

### 2.4. Analysis

To process the data for statistical analysis, we imported the eye-tracking data from the Tobii X2-30 Compact into Microsoft Excel. Each image presented to the participant would generate roughly 5000 milliseconds of data for each AOI, either shown as 0 (no eye-gaze behaviour detected in the AOI) or 1 (eye-gaze behaviour detected in the AOI). For each fixation event, duration was calculated as the difference between the earliest and latest recording time stamps. Total fixation duration for each AOI was then calculated as the sum of all fixation events per AOI and participant. This was repeated for all AOIs for all 30 images for each participant. The number of fixations was calculated by counting every fixation for each AOI for each image and participant. Data were analysed using SPSS, version 28 (Statistical Package for the Social Sciences). The planned analysis strategy was a repeated measures MANOVA; however, after checking, it became clear that the assumptions of this model were violated (for example, no dependent variable was normally distributed). Therefore, linear mixed models were used instead, with dependent variables nested within participants, and rating type (attractiveness, healthiness, and youthfulness), and model sex, model BMI, and AOI included as fixed factors. Separate models were run with fixation duration, number of fixations, and Likert ratings as dependent variables.

## 3. Results

### 3.1. Eye-Gaze Behaviour

Main effects and all interaction terms between the dependent variables were included initially. All three and four-way interaction terms were non-significant, so they were not included in the final model. Table 1 reports the main effects and two-way interactions.

**Figure 3:**
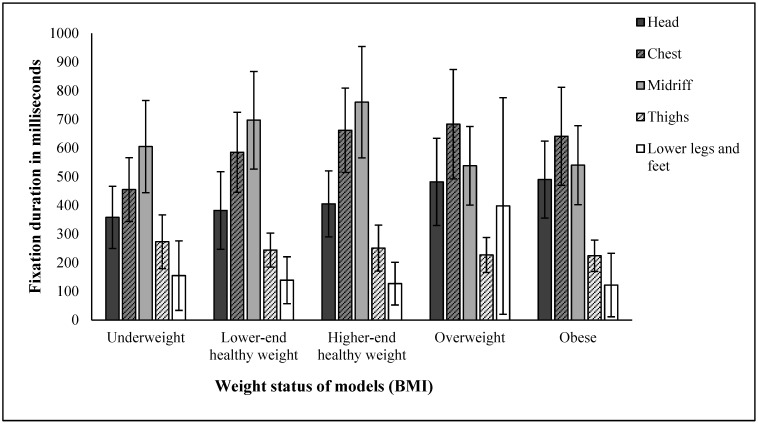
Total fixation time across AOI and BMI. Error bars are 95% CIs.

**Figure 4:**
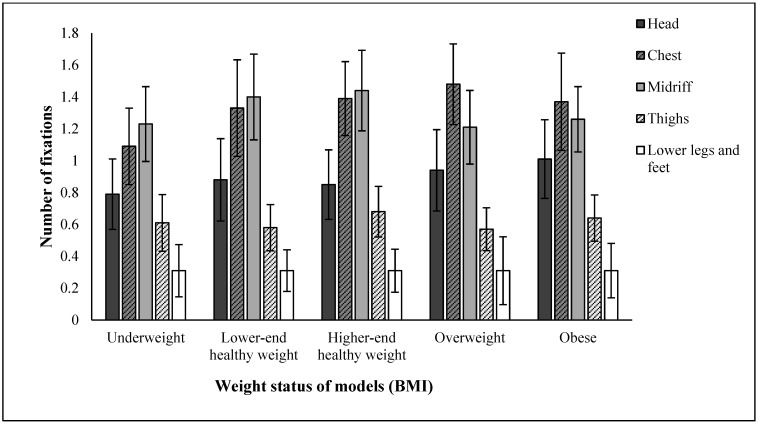
Number of fixations across AOI and BMI. Error bars are 95% CIs.

AOI and sex had significant effects on fixation duration and numbers of fixations, but not BMI or rating type. Unsurprisingly, participants looked at some AOIs more than others, with most visual attention paid to the chest and midriff, and the least to the thighs and lower legs and feet. More interesting was that participants looked more at male vs. female stimuli both in terms of duration (2178 ms vs. 1895 ms across all AOIs) and number of fixations (4.8 vs. 4.1). Model BMI and AOI had a significant two-way interaction, whereby the participants’ fixation duration at specific areas of the body were significantly different depending on the model’s body size (Figure 3 and Figure 4). For example, the head received more attention as BMI increased, while the midriff received the most attention at intermediate BMIs.

**Figure 5:**
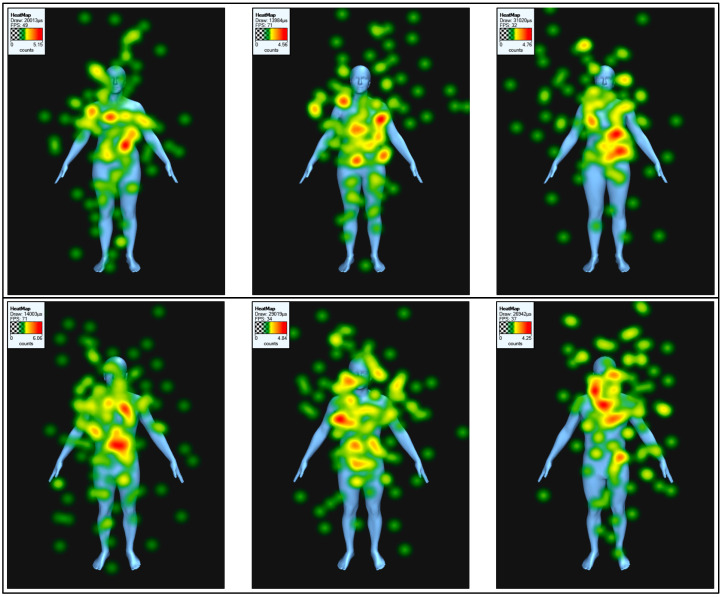
Heat maps showing eye-gaze behaviour on three higher-end healthy weight female (**top**) and male (**bottom**) models when rating attractiveness (**left**), healthiness (**middle**), and youthfulness (**right**).

Rating type itself had no effect on gaze behaviour, but it did interact with AOI, whereby different areas of the body were fixated more or less depending on what rating was being made (Figure 5). Differences are subtle, but healthiness ratings focus more on the chest and midriff, while youthfulness ratings involve more attention to the head.

There was a significant interaction between model sex and rating type on the number and duration of fixations, such that participants looked for longer when judging attractiveness in male stimuli, but not female (Table 2).

There was a significant interaction between model sex and AOI on the number of fixations (but not duration), such that participants made more fixations on different areas in men and women (Table 3).

The interaction between BMI and rating type was significant for the number of fixations but not duration, such that the most fixated size model varied depending on the rating being made (Table 4). For example, participants fixated the most times at the higher-end healthy weight model and for the shortest at the underweight model when judging attractiveness, whereas gaze behaviour was more even across the models when judging healthiness or youthfulness. The interaction between model sex and BMI was not significant.

Post hoc power analysis using G*Power (v 3.1.9.7; Faul et al., 2007) indicated that our sample size of 32 participants gave 100% power to detect the largest multivariate effect of *η*^2^*_p_* = 0.306 and 19% power to detect the smallest effect of *η*^2^*_p_* = 0.014. This sample size had 80% power to detect an effect of *η*^2^*_p_* = 0.064.

### 3.2. Likert Ratings

A linear mixed model was run with Likert ratings nested within participants, and fixed factors of sex and BMI of the models and rating type (Table 5).

The main effects of BMI and rating type on Likert scores were strong and significant, while the effect of model sex was marginally significant. The interaction between model sex and rating was significant, indicating that attractiveness, healthiness, and youthfulness were judged differently on the male and female models (Table 6). Although model sex significantly affected perceived healthiness scores (females were rated higher than males), there was no significant effect on perceived attractiveness and youthfulness scores.

**Figure 6:**
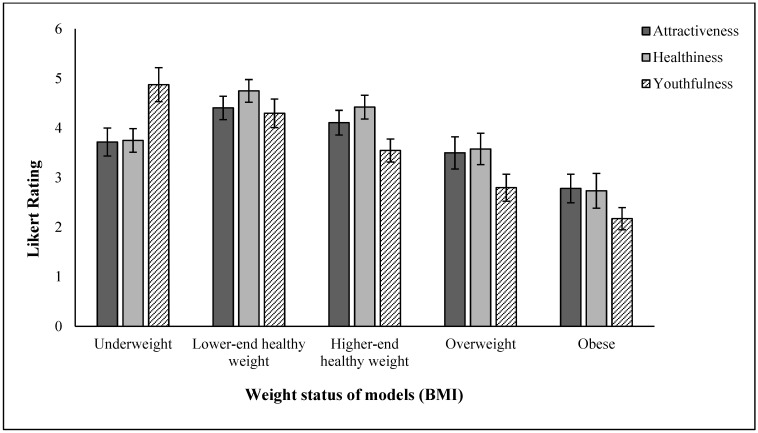
Ratings of attractiveness, healthiness, and youthfulness for models of various weight status (BMI). Error bars are 95% CIs.

The interaction between rating and BMI was significant, indicating that the effect of BMI was different for the different types of ratings, as was the three-way interaction, indicating that this pattern differed depending on the sex of the model (Figure 6).

**Figure 7:**
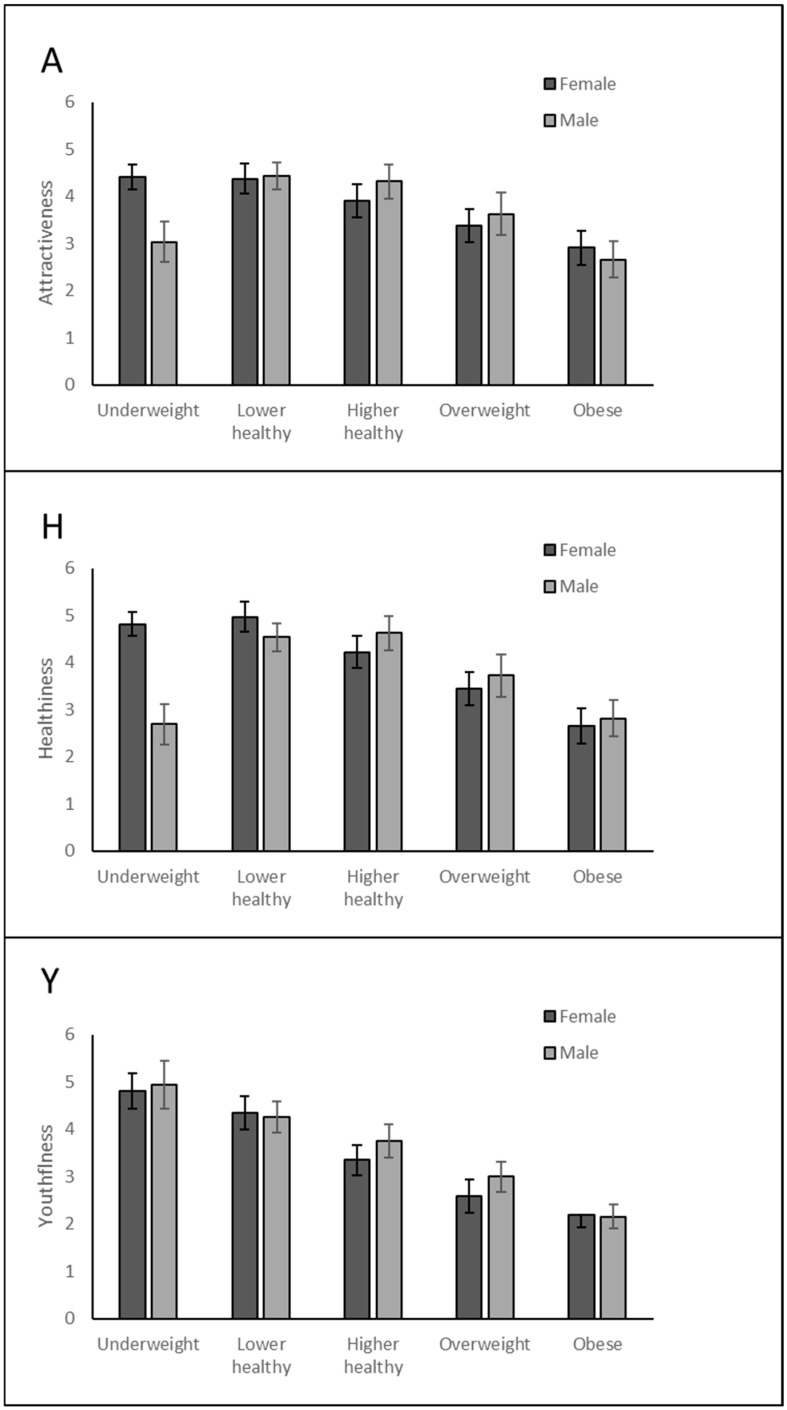
Ratings of attractiveness (A), healthiness (H), and youthfulness (Y) of female and male models across BMI. Error bars are 95% CIs.

The interaction between model sex and BMI was significant, indicating that the effect of BMI on Likert scores was different for male and female models. Furthermore, the three-way interaction between model sex, BMI, and rating was also significant, indicating that the interaction above varied depending on the type of rating being made. For example, youthfulness had a linear decrease with BMI in both sexes, whereas male attractiveness and healthiness were also low at underweight BMI, but not for these female ratings. These patterns are illustrated in Figure 7.

## 4. Discussion

### 4.1. Findings and Implications

This study suggests that people look at different parts of the body depending on their sex, BMI, and the type of judgement they are making. The most basic, and most unsurprising finding, was that most visual attention was paid to the chest and midriff, followed by the head, with the least attention paid to the legs. This pattern is similar to previous research (e.g., Cornelissen et al., 2009); however, Dixson et al. (2011) and Hewig et al. (2008) found most attention paid to the head more than the chest and midriff, probably because their models were more realistic images with facial features, compared with the relatively featureless faces on ours. We also found that more attention was paid to male rather than female models, possibly because most of our participants were heterosexual women for whom the male bodies were most mate-choice relevant. This is supported by the finding that participants looked for longest when judging male attractiveness rather than healthiness or youthfulness.

The more interesting findings were that the parts of the body that were fixated changed with BMI. At the underweight and healthy weights, the most visual attention was paid to the midriff. At overweight and obese levels, the most attention was paid to the chest instead. Dixson et al. (2011) speculate that attention to women’s breasts and buttocks may be because these are areas of secondary-sexual fat deposition, which may indicate sexual maturity and attractiveness. Judgement type did not by itself affect gaze behaviour, suggesting overall visual attention was the same when making different ratings, but it did affect where people looked, with attention more evenly distributed across head, chest, and midriff when judging youthfulness, whereas the head was less fixated when judging attractiveness or healthiness. This may be because the face typically strongly indicates maturity, so even though our models were relatively featureless, people may have looked at the face automatically when judging youthfulness. This pattern of results is consistent with previous literature, which has found that participants tended to fixate on the upper half of the body (central and upper abdomen and chest) when making attractiveness judgements and body fat judgements on female stimuli (Cornelissen et al., 2009). Judgement type also affected how attention was paid to different body sizes, with a peak in visual attention at the high healthy weight when judging attractiveness, but not healthiness or youthfulness, where attention was more evenly distributed. We speculate that this might be because the male model BMI 24.5 may most closely resemble a “mesomorph” build, i.e., muscular but quite lean. Such mesomorph bodies are rated most attractive and receive the most visual attention (Dixson et al., 2014).

The sex of the model also affected eye gaze, with more visual attention paid to male models when judging attractiveness and healthiness compared with youthfulness, a pattern not seen with female models. This may be because the male models gave more cues relevant to attractiveness and healthiness, such as musculature (Franzoi & Herzog, 1987). This is supported by the finding that more fixations were given to the chest and midriff of the male models than the female ones, and these areas contain muscles in the shoulder, chest, and abdominal area. Such upper body musculature is a strong predictor of bodily attractiveness (Sell et al., 2017).

BMI had the biggest effect on ratings of attractiveness, healthiness, and youthfulness, in line with previous research on attractiveness (Tovée et al., 1999a) coupled with the fact that BMI is associated with poorer health (Pi-Sunyer, 1999) and it tends to increase with age (Ogden et al., 2006; Sakamoto, 2006). These results support the health and fertility hypothesis, which states that preferred and “more attractive” leaner bodies are perceived to be healthier (Tovée et al., 1999a, 1998). This implies that people generally find healthy body sizes attractive. On the other hand, the present results are inconsistent with C. Han et al.’s (2023) recent work on testing the health and fertility hypothesis. The key findings of their study were that the participants’ (male) perceived most attractive female models had a lower BMI than the participants’ perceived healthiest models. These results implied that the participants knew their preferred attractive model was not necessarily the healthiest. In contrast, our current results implied that the “attractive” model chosen by our participants was consistent with a “healthy” body size. This difference could be due to cultural differences since Han et al. utilised a sample of Chinese participants. The current sample, however, comprised Western (mainly British/European origin) participants. Since Asian countries have lower BMI categories than Western countries, the previous study participants may have experienced harsher societal norms and therefore associated lower BMIs as attractive but not healthy. There is evidence that body-size preferences vary substantially across cultures (Boothroyd et al., 2020; Tovée et al., 2006), and so cross-cultural replication would be valuable to establish the similarity or differences in both ratings and eye-gaze behaviour made on bodies of varying size.

The pattern of ratings was slightly different for men and women, with underweight men rated much lower on attractiveness and healthiness, whereas underweight women were still rated high. This is probably because underweight men have very low musculature, which is unattractive, as discussed above, whereas women’s musculature is lower overall and is not so strongly linked to attractiveness. Furthermore, the underweight BMI of 17.5 was only just below the healthy threshold of 18. The website used to create the models does not allow visualizations below this weight, but if we had used even more underweight models (e.g., BMI of 15), we may well have found these were rated less attractive and healthy in both sexes. This pattern also reinforces the fact that leanness is idealised more in women’s bodies than in men’s (Ahern et al., 2011). Crossley et al.’s (2012) study pinpointed ideal female BMI as 18.8–18.9, but 24.5–25.9 for male models, in a similar pattern to our study.

### 4.2. Limitations and Future Directions

Our study is limited in its ecological validity in that it used models merely resembling female and male humans rather than real people. Previous findings have discovered a significant change in results when layouts of stimuli were altered, therefore advising researchers to expressly state their layout choices for replicability purposes (Spinner et al., 2013). A recent study combatted this issue by utilising eye-tracking equipment in unconstrained natural settings, examining the participants’ eye-gaze behaviour when perceiving others passing by in the street (Varela et al., 2023). This would provide an excellent foundation for future eye-tracking research. However, using actual photographs or real-life scenarios loses the control of computer models, in which body size and shape can be controlled precisely, allowing experimental manipulation and strong interpretation of cause and effect. Another limitation is that our sample consisted of mostly female participants. Prior research has found sex differences in eye-gaze behaviour (Garza et al., 2016; Rupp & Wallen, 2008). Future research with a sample of an equal number of male and female participants may provide more valid results and allow testing for sex differences in perception.

Because we used models of varying size, it is important to note that the size of different AOIs was not entirely consistent, i.e., larger models had larger AOIs. However, BMI itself did not affect overall gaze behaviour, suggesting that this artefact did not confound results. Furthermore, in real life, larger people do occupy more visual space and so may capture more visual attention. A further limitation is that our order of ratings was fixed and, therefore, may have resulted in order effects. Future studies could randomise the order of presentation when using multiple ratings. Participants’ own body sizes might also be related to how they look at and judge others, perhaps through “norm comparison,” whereby other bodies are compared with one’s own (Robinson & Kersbergen, 2017); therefore, measuring participants’ BMI would be a useful addition to future research.

### 4.3. Conclusions

This study expands the field of human social perception by using eye-tracking to objectively measure visual attention. It adds to previous eye-tracking research by including the judgements of healthiness and youthfulness, which are theorised to be close to attractiveness. The finding that eye-gaze behaviour changed depending on the judgement being made suggests these judgements are similar but subtly different, so perhaps partly independent.

## Figures and Tables

**Table 1:** Linear mixed-effect models showing main effects and interactions on fixation duration and the number of fixations.

Fixed Effects	Fixation Duration (*F*(*df*), *p*)	Number of Fixations (*F*(*df*), *p*)
Main effects		
Model sex	***F*(1,4746) = 12.14, *p* < 0.001**	***F*(1,4746) = 26.65, *p* < 0.001**
BMI	*F*(4,4746) = 1.95, *p* = 0.100	*F*(4,4746) = 3.00, *p* = 0.057
Rating	*F*(2,4746) = 0.55, *p* = 0.578	*F*(2,4746) = 0.74, *p* = 0.479
AOI	***F*(4,4746) = 139.98, *p* < 0.001**	***F*(4,4746) = 180.90, *p* < 0.001**
2-way interactions		
Model sex × BMI	*F*(4,4746) = 1.39, *p* = 0.236	*F*(4,4746) = 2.16, *p* = 0.070
Model sex × rating	***F*(2,248) = 4.89, *p* = 0.008**	***F*(2,248) = 3.87, *p* = 0.021**
Model sex × AOI	*F*(4,4746) = 1.72, *p* = 0.141	***F*(4,4746) = 3.17, *p* = 0.013**
Rating × BMI	*F*(8,4746) = 1.35, *p* = 0.212	***F*(4,4746) = 2.46, *p* = 0.012**
BMI × AOI	***F*(16,4746) = 2.79, *p* < 0.001**	*F*(8,4746) = 1.31, *p* = 0.178
Rating × AOI	***F*(8,4746) = 2.89, *p* = 0.003**	***F*(16,4746) = 2.58, *p* = 0.008**

Effects significant at *p* < 0.05 are in bold.

**Table 2:** Interaction between sex and rating type on fixation duration.

Rating	Female	Male
Fixation duration (ms)		
Attractiveness	363 (286–441)	473 (395–550)
Healthiness	369 (298–440)	444 (363–524)
Youthfulness	403 (327–479)	391 (311–470)
Number of fixations		
Attractiveness	0.79 (0.65–0.94)	1.04 (0.90–1.12)
Healthiness	0.80 (0.64–0.96)	0.96 (0.81–1.10)
Youthfulness	0.86 (0.70–1.00)	0.90 (0.73–1.08)

Data are mean (95% CI).

**Table 3:** Interaction between sex and AOI on number of fixations.

AOI	Female	Male
Head	0.83 (0.62–1.04)	0.96 (0.73–1.19)
Chest	1.21 (0.97–1.45)	1.46 (1.20–1.71)
Midriff	1.16 (0.96–1.36)	1.45 (1.21–1.70)
Thighs	0.57 (0.45–0.70)	0.66 (0.51–0.81)
Lower legs	0.31 (0.16–0.46)	0.31 (0.16–0.47)

Data are mean (95% CI).

**Table 4:** Interaction between BMI and rating on gaze behaviour.

	Attractiveness	Healthiness	Youthfulness
# fixations			
Underweight	0.77 (0.58–0.94)	0.85 (0.67–1.03)	0.80 (0.62–0.98)
Low healthy	0.85 (0.69–1.02)	0.99 (78–1.20)	0.86 (0.65–1.07)
High healthy	1.07 (0.92–1.22)	0.88 (0.71–1.04)	0.86 (0.68–1.04)
Overweight	0.90 (0.75–1.06)	0.81 (0.63–0.99)	0.99 (0.81–1.17)
Obese	0.99 (0.93–1.14)	0.87 (0.68–1.05)	0.89 (0.74–1.04)

Data are mean (95% CI) and are averaged across both sexes.

**Table 5:** Linear mixed-effect model showing the effect of model sex, BMI, and rating on Likert scores.

Fixed Effects	Likert Score
Main effects	
Model sex	*F*(1,930) = 3.79, *p* = 0.052
BMI	***F*(4,930) = 122.37, *p* < 0.001**
Rating	***F*(2,930) = 8.30, *p* < 0.001**
2-way interactions	
Model sex × BMI	***F*(4,930) = 19.25, *p* < 0.001**
Model sex × rating	***F*(2,930) = 5.79, *p* < 0.001**
Rating × BMI	***F*(8,9306) = 15.02, *p* < 0.001**
3-way interaction	
Mode sex × BMI × rating	***F*(8,930) = 4.73, *p* < 0.001**

Effects significant at *p* < 0.05 are in bold.

**Table 6:** Ratings of male and female models across BMI.

Rating (1–6)	Female	Male
Attractiveness	3.79 (3.51–4.07)	3.61 (3.37–3.85)
Healthiness	4.02 (3.77–4.23)	3.68 (3.47–3.88)
Youthfulness	3.46 (3.22–3.69)	3.62 (3.42–3.82)

Data are mean (95% CI).

## Data Availability

The data supporting the conclusions of this article are available on reasonable request from the authors.
